# Blindesss in Texas

**Published:** 1894-08

**Authors:** H. L. Hillgartner

**Affiliations:** Austin


For Texas Medical Journal.

### BLINDNESS IN TEXAS.

BY H. L. HILGARTNER, M. D.

Read at the meeting of the State Medical Association, April 26, 1894.

MR. PRESIDENT AND GENTLEMEN:—Research of late years, pursued in one direction, has revealed a condition of things which concerns not alone the eye-specialist and the medical profession, but every one who considers our national welfare. To the consideration of the alarming increase of blindness in the United States—an increase out of all proportion to the simultaneous growth of population—I ask your attention.

In 1887, Dr. Lucien Howe (Transactions of the American Ophthalmological Society, 1887,) presented a paper at the meeting of the American Ophthalmological Society in which he called attention to the fact that, according to the census reports, the number of blind in this country was increasing much more rapidly than the population. His figures, taken from the United States census of 1870–1880, show an increase of 30.09 per cent. in the population for the decade of 1870–1880, and 140.78 per cent. in the number of the blind. It was also shown that the rate of blindness increased from North to South and decreased from East to West. The conclusions arrived at by Dr. Howe were the following:

Blindness is greater, in proportion to the population, in the Eastern States; that it is proportionately greater in these States in the thickly populated, than in the rural districts; that the crowding together of children in institutions seems to be a prolific cause; that contagious eye diseases are often brought in by immigrants; that the financial loss to the nation is immense, from the necessity of supporting this great number of blind persons.

These conclusions led the Ophthalmological Society to appoint a committee to examine into the subject, as did also the Medical Society of the State of New York, after the same figures had been presented there for consideration. The committee, in both of these societies, reported the advisability of some legislation to limit the further increase of what may be considered one of the principal causes of blindness, namely, ophthalmia neonatorum. Since that time the States of New York, Maine and Rhode Island have passed laws which have for their object the preventive treatment of this disease. In the remainder of this paper I hope to show the desirability of a similar law in our own State.

The investigations made in New York show an increase in the blindness in this State, between 1870–1880, of 125.7 per cent., with a simultaneous increase of population of 15.9 per cent. Accordingly blindness in New York State had increased 8.2 times as rapidly as had the population. At the meeting of the Medical and Chirurgical Faculty of Maryland, held at Baltimore in April, 1891, Dr. Woods (*Maryland Medical Journal*, May 9th, 1891), of Baltimore, read a carefully prepared paper in which he stated an increase in the population of about 20 per cent., and an increase of 121 per cent. in number of the blind for the period between 1870–1880. Further observations showed an apparent increase of 95 per cent. in the blind of ages from 4 to 24 years, for the time between 1880 and 1890. These figures were the result of personal examination of pupils at the Maryland school for the blind.

Dr. Woods stated that the last figures could not be so relied upon as those for the preceding ten years. However, the result is sufficient to show the enormous increase of blind in proportion to the increase in population for the State of Maryland.

The population in Texas, in 1880, was 1,691,740; in 1890, the census gave us 2,236,623, an increase of 40.2 per cent. In 1880, we had 1349 blind; in 1890 the blind numbered 1560. We find an increase of only 15 per cent.

The census of 1890 gives us the following figures:

Blind in the United States in 1890, 50,411.

Blind in the United States in 1880, 48, 929.

We find an increase for the entire United States of only 3.20 per cent. Considering the last census, a less complete record of such persons than the census of 1880, the important fact, that a very large percentage of the blindness is caused simply by neglect in early infancy, is still apparent.

For a moment let us consider the various causes of blindness and note conclusions arrived at from the investigations made at the State Blind Institutions. In order to make comparisons, we will first glance at the causes of blindness in New York.

The committee of the Ophthalmological Society (Transactions American Ophthalmological Society, 1890,) were able to get at the exact conditions of the eyes of 509 individuals of all ages. The following are a few of the summary notes:

2.27 per cent. was congenital; 14.51 per cent. was due to ophthalmia neonatorum; 7⅞ per cent. was caused by trachoma or granular lids; 12-51 per cent. came from primary or secondary corneal diseases, and 8.21 per cent. from sympathetic ophthalmia.

According to above figures 43.01 per cent. of the blindness in New York State came from four diseases. Of these ophthalmia neonatorum and sympathetic ophthalmia are wholly preventable, while trachoma and corneal diseases are curable in most instances, if proper treatment be instituted in the early stage of the diseases.

In order to ascertain causes of blindness in Texas, I examined the pupils at the State blind schools. I experienced great difficulty in obtaining histories of these cases, on account of ignorance manifested by many of the pupils, and carelessness of parents. Indeed, in a large number, sole reliance was put upon objective appearance.

At the white school I examined 167 pupils, their ages ranging from 7 to 27. Of this number, 15 per cent. were blind from ophthalmia neonatorum; 5.1 per cent. from sympathetic ophthalmia;

10.7 per cent. from trachoma. At the colored school 46 cases were examined; their ages ranged from 7 to 29. Four were blind from sympathetic ophthalmia; 5 from trachoma (3 of which were brothers); 8 from ophthalmia neonatorum; 1 from gonorrhœal ophthalmia.

Of these diseases, all of which are almost entirely preventable, there is one, "ophthalmia neonatorum," which I wish to consider at some length. Ophthalmia neonatorum, also known as "babies sore eyes," is the cause, according to Magnus, of 23.5 per cent. of blindness in Europe among persons under 20 years of age; of 10.87 per cent. *of all* blindness; of 14.51 per cent. of blindness in New York State; of 17.6 per cent. in Maryland; 15 per cent. in Texas. This disease is considered entirely preventable and curable—certainly in 98 per cent. of all cases.

The important questions which present themselves are:

1. Why does ophthalmia neonatorum continue to cause so much blindness?

2. What can be done to prevent further increase in blindness from this source?

The answer to the first question, as given by Dr. Woods, "is ignorance; ignorance of its great danger on the part of parents; ignorance on the part of mid-wives, and too often ignorance on the part of medical attendants."

As to the second question, we must consider what means can be adopted to bring these children, as soon as possible, to the notice of a competent physician. Education of the laity is useless; urging nurses is equally insufficient. The surest and best means of accomplishing this is undoubtedly by legislation. This was the view taken of the subject by those who have considered it most carefully, and following the plan which had been partially adopted before in Switzerland,—a concise but explicit bill for the proper protection of these infants, was passed by both houses of the New York legislature, 1890, without a dissenting vote, and became a law at once. The State of Maine was the second to pass such a law, and Rhode Island the third State to have such a law. There is a similar bill before the legislature of Ohio at the present time.

The disease is rarely seen outside the circles of the poor. The problem to solve is how to protect babies of the poor; children born under the care of medical men who do not realize the dangers of infantile ophthalmia, or of mid-wives who may have never heard of it. The Eye Infirmary of Sheffield, England, distributes among the poor, by means of the poor physicians, the following card:

IMPORTANT NOTICE: If a baby's eyes run with matter and look red a few days after birth, take it *at once* to a doctor.

*Delay is dangerous*, and one or both eyes may be destroyed if not treated *immediately*.

The distribution of such cards by the health authorities would undoubtedly accomplish much good. The question in connection with mid-wives is the most difficult to answer. Our laws require nothing but self-confidence on the woman's part to entitle her to license. These women attend many more poor women in confinement than do physicians, and are naturally interested in covering up their mistakes. In various parts of Europe, Saxony, Austria, Prussia, etc., there are laws of more or less stringency regulating the licensing of these women, compelling them to know Credes' method before obtaining their licenses, and to use it in practice.

A law of this kind makes a nurse appreciate her responsibility and lets her know, that the condition indicated by the redness and discharge in the newly born is not to be trifled with.

Having shown that such legislation should be enacted, it is, Mr. President, our duty to consider this matter at once, and take the necessary steps which will have for their result the adoption of a suitable law in the State of Texas.

